# Adolescent Sleep Patterns and Night-Time Technology Use: Results of the Australian Broadcasting Corporation's Big Sleep Survey

**DOI:** 10.1371/journal.pone.0111700

**Published:** 2014-11-12

**Authors:** Amanda L. Gamble, Angela L. D'Rozario, Delwyn J. Bartlett, Shaun Williams, Yu Sun Bin, Ronald R. Grunstein, Nathaniel S. Marshall

**Affiliations:** 1 NHMRC Centre for Integrated Research and Understanding of Sleep (CIRUS), The University of Sydney, Sydney, NSW, Australia; 2 Sleep and Circadian Research Group, Woolcock Institute of Medical Research, Sydney, NSW, Australia; 3 Department of Sleep and Respiratory Failure, Royal Prince Alfred Hospital, Sydney, NSW, Australia; 4 Sydney Nursing School, The University of Sydney, Sydney, NSW, Australia; University of Perugia, Italy

## Abstract

**Introduction:**

Electronic devices in the bedroom are broadly linked with poor sleep in adolescents. This study investigated whether there is a dose-response relationship between use of electronic devices (computers, cellphones, televisions and radios) in bed prior to sleep and adolescent sleep patterns.

**Methods:**

Adolescents aged 11–17 yrs (n = 1,184; 67.6% female) completed an Australia-wide internet survey that examined sleep patterns, sleepiness, sleep disorders, the presence of electronic devices in the bedroom and frequency of use in bed at night.

**Results:**

Over 70% of adolescents reported 2 or more electronic devices in their bedroom at night. Use of devices in bed a few nights per week or more was 46.8% cellphone, 38.5% computer, 23.2% TV, and 15.8% radio. Device use had dose-dependent associations with later sleep onset on weekdays (highest-dose computer adjOR  = 3.75: 99% CI  = 2.17–6.46; cellphone 2.29: 1.22–4.30) and weekends (computer 3.68: 2.14–6.32; cellphone 3.24: 1.70–6.19; TV 2.32: 1.30–4.14), and later waking on weekdays (computer 2.08: 1.25–3.44; TV 2.31: 1.33–4.02) and weekends (computer 1.99: 1.21–3.26; cellphone 2.33: 1.33–4.08; TV 2.04: 1.18–3.55). Only ‘almost every night’ computer use (: 2.43: 1.45–4.08) was associated with short weekday sleep duration, and only ‘almost every night’ cellphone use (2.23: 1.26–3.94) was associated with wake lag (waking later on weekends).

**Conclusions:**

Use of computers, cell-phones and televisions at higher doses was associated with delayed sleep/wake schedules and wake lag, potentially impairing health and educational outcomes.

## Introduction

Poor sleep is a common and debilitating problem of adolescence, affecting around 25–40% of teenagers at some point in their development [1], [2]. The onset of puberty triggers hormonal changes that delay circadian rhythms producing a physiological drive toward later sleep and wake times [3]. At the same time, adolescents become less sensitive to the build-up of homeostatic sleep pressure allowing them to stay awake for longer periods [4]. These changes are often incompatible with societal demands that require teenagers to start school early, resulting in sleep restriction throughout the school week and a tendency to wake later on weekends to ‘catch up’ on lost sleep [5]. The resulting misalignment between weekday and weekend sleep schedules, exacerbates the underlying sleep deficit [6], [7]. Other psychosocial influences such as increased workload [8] and greater autonomy in setting bedtimes [9], further contribute to the risk of poor sleep, ultimately resulting in sleepiness and fatigue [10], impaired academic performance [11], and potentially placing adolescents at increased risk of anxiety, depression and substance abuse [12], [13], obesity [14], [15], diabetes [16], and cardiovascular disease [17].

In recent years, the proliferation of electronic devices (EDs) such as computers and cellphones has been implicated in the poor sleep of young people. Surveys have linked the mere presence of EDs in the bedroom with later bedtimes, less time in bed, shorter sleep duration and daytime sleepiness [18], [19], [20], prompting widespread recommendation that EDs be removed from the bedroom. However, it is the actual use of EDs that is of greatest clinical relevance. US Adolescents are heavy users of EDs - 72% report using cellphones, 60% laptops and 23% video games in the hour prior to sleep [21]. A 2010 review of 36 youth studies from around the world linked use of such devices prior to sleep with late sleep and wake times and short sleep duration [22]. Night time technology use is also linked with functional impact including increased sedentary behaviour prior to bed [23], subjective poor sleep quality [24], greater caffeine consumption, falling asleep in school and increased daytime sleepiness [25].

There are two issues which require further exploration. First, studies have not yet examined the degree of usage likely to impact sleep in young people (i.e. the dose-response curve). This could aid development of real world-applicable guidelines for ED use in young people as complete exclusion of devices from the sleeping environment no longer seems feasible or acceptable as a blanket approach. Second, little is known about ED use as it relates to irregular weekday vs. weekend sleep schedules. Prior studies of ED use have tended to examine sleep patterns averaged across the entire week, overlooking the tendency of young people to wake later and to extend sleep duration (i.e., catch up sleep) on weekends [7]. This study examines whether there are dose-response relationships between ED use in bed prior to sleep and the likelihood of ‘problematic sleep’ including: i) late sleep onset and wake times on weekdays (sleep onset after midnight, waking after 8 am) and weekends (sleep onset after 1 am, waking after 10:30 am); ii) short sleep duration on weekdays (less than 8 hrs) and weekends (less than 9 hrs); and iii) an increased tendency to wake later by more than 2.5 hrs on weekends relative to weekdays (i.e., wake lag) and extend sleep duration by more than 1.5 hrs on weekends (i.e., catch-up sleep). This behavioural definition of ‘problematic sleep’ was derived from the findings of a recent meta-analysis of worldwide trends in adolescent sleep patterns [26]; combined with social constraints known to influence adolescent sleep patterns such as Australian school start times [27] (see Methods for greater detail).

## Methods

The adolescent participants that are the subject of this particular report gave their own written consent to participate. The University of Sydney Human Research Ethics Committee provided ethics approval for adolescent participants to provide their own consent rather than requiring consent from next of kin, caretakers or guardians and to analyse data obtained after 12th August, 2010 and only these data are presented (protocol number 12590).

The Big Sleep Survey was a web-based survey of Australian sleep habits undertaken by the Australian Broadcasting Corporation (ABC; a government-funded media organisation akin to the BBC in the United Kingdom or the PBS in the United States) in conjunction with the NHMRC Centre for Integrated Research and Understanding of Sleep (CIRUS) at the University of Sydney. The survey was conducted as part of Australian National Science Week 2010, and participants were recruited via ABC national media (internet, television and radio) advertisement. The survey was available online for participants to complete from late August 2010 until January 2011 (almost all data accrued in the first few weeks), coinciding with Australian spring and summer and little seasonal variation in sunlight across the continent's Longitude (113° E and 153°E) and Latitude (11°S and 38°S). An electronic consent form was displayed prior to entering the survey. Participants did not receive reimbursement but were incentivised by entry into a draw to win an Apple iPad. After completing the survey, participants were provided with feedback about their sleep habits and were directed to seek assessment where responses indicated sleep problems.

### Demographics and Self-Reported Sleep Problems

The survey consisted of demographics, medical and psychiatric history, factors affecting sleep such as exercise, smoking, caffeine intake, alcohol use, medication use, shift work, and sleep disorders and their treatments. Participants self-reported their average: 1) sleep duration [How many hours of sleep would you normally get (excluding naps)?]; 2) number of awakenings [On average, how many times do you wake up during the night?]; and 3) sleep onset latency [How long does it take you to fall asleep? (Select from 0–5 mins, 5–15 mins, 15–30 mins, or More than 30 mins)].

### Weekday vs. Weekend Sleep Schedules

Analyses were based on self-reported sleep duration, and sleep onset and wake times, which were derived from participants' responses to the following questions: On a typical ‘working’ day (for most people this will be weekday), what time would you: 1) wake up; 2) go to bed; and 3) go to sleep. These questions were repeated for a typical non-working day (i.e., the weekend). Wake lag was calculated as the difference between wake times on school days vs. weekends. Catch up sleep was calculated as the difference between sleep duration on weekdays and weekends. The categorical handling of these data are described in the statistical analyses and data handling section below.

### Presence and Use of Electronic Devices

Participants indicated the presence (yes/no) of the following devices in their bedroom: television; computer or laptop; mobile phone (i.e., cellphone, pager or Blackberry); and radio. Participants were asked “During the hours you would normally be sleeping how often would you do the following activities in bed?” they then rated how often they performed each of the following on a 5-point likert scale: a) Watch television; b) Listen to the radio; c) Use the computer or laptop; and d) Use your mobile telephone and/or pager.

Frequency of ED use was rated on this 5-point Likert scale (Never, Rarely, A Few Nights a Month, A Few Nights a Week, Every or Almost Every Night). The wording of this question meant that the duration of device use in bed and the time-scale over which they might refer their behaviour (days, weeks or for instance months) was self-defined for each participant. For the purpose of analysis, those that ‘Never’ used the device were further divided into 2 subgroups that reported the device being absent vs. present in the bedroom. This created a 6-category ordinal ‘dose’ of ED use for logistic regression analyses. The purpose of this subdivision was to investigate whether a device being present in the bedroom represented any additional risk over nil usage.

### Other Measures

The Epworth Sleepiness Scale [28] asked about propensity to fall asleep in everyday situations. Socioeconomic status was estimated using the Index of Relative Socio-economic Advantage and Disadvantage (IRSAD) derived from Socio-Economic Indexes for Areas (SEIFA) Australian census data [29]. IRSAD is comprised of 21 measures including low or high income, internet connection, occupation and education. The national average is 1000 and the SD is 100. Higher values indicate more advantaged areas.

### Statistical Analyses and Data Handling

Paired t-tests were performed to compare sleep duration, and sleep onset and wake times on weekdays vs. weekends. Effect sizes were calculated using Cohen's d. Multivariate logistic regression models were performed to examine dose relationships between the predictor variable (frequency of ED use in bed when participants would normally be sleeping) and problematic sleep patterns. Logistic models were first calculated using dose as a categorical variable to calculate the odds ratios plotted in the figures and table S1–S8 in File S1. We then ran the same models with dose as an ordinal variable to derive the p-value in the top left hand corner of all of the outcomes figures which we used to test for the presence of a dose-response pattern. We built our definitions of ‘problematic sleep’ from two sources: 1) a meta-analysis of worldwide adolescent sleep/wake trends, which set the cut-off for sufficient sleep at 8 hours, and reported average wake lag to be 2.5 hours and average catch up sleep to be 1.5 hours [26]; and 2) practical determinants of adolescent sleep schedules such as Australian school start times of 8:30 am. For example, an Australian adolescent who intends to obtain at least 8 hours sleep on a school-night must logically establish sleep onset by midnight and wake by 8 am at the latest in order not to be truant from school. Accordingly, problematic sleep on weekdays was defined as: sleep onset occurring after midnight, waking after 8:00 am, and obtaining less than 8 hours sleep. Problematic sleep on weekends was defined as: sleep onset occurring after 1 am, waking after 10:30 am, and obtaining less than 9 hours sleep. Waking up more than 2.5 hours later on weekends relative to weekdays was considered abnormal wake lag (greater than observed average of 2 hours and 7 mins). Extending sleep duration by more than 1.5 hours on weekends relative to weekdays was considered abnormal catch-up sleep (greater than observed average of 1 hour and 23 mins). Multivariate analyses controlled for age, gender, socioeconomic status and caffeine consumption as core confounders. Caffeine consumption was placed into quartiles to limit the effect of outliers and positive skew. Because of heavy clustering at the median the above cut-offs coincided with the worst two quintiles of each variable rather than the median. In our logistic regression models we also conducted sensitivity analyses using a worst 10% cutoff and these did not change our conclusions. To control for the number of analyses, significance was defined at *α*<.01 and we report 99% confidence intervals throughout.

We have presented all of our analyses for each exposure and all outcomes in the same manner in each of the figures so that readers can accurately judge the likelihood for type 1 error rather than construct a narrative ‘just so’ story around a handful for positive associations. We are also forced into analyzing all of the 8 sleep behavior outcomes because there is no consensus about what parts of these sleep behaviors are actually important (or redundant) when assessing health or social effects.

## Results

### Demographics and Self-Reported Sleep Problems

A total of 11,451 individuals (aged 11–93 years) who reported Australian zip-codes completed the survey (total responses including overseas addresses was 11,797). These analyses focus on data from 1,184 respondents that met inclusion criteria, that is, being community-dwelling high-school students aged 11–17 years. Ninety nine per cent of data were collected during the school term. Demographics and self-reported sleep problems are shown in Table 1. In the week prior to completing the survey, participants consumed an average of 5.04 caffeinated drinks, however data were positively skewed, and therefore median caffeine use (and interquartile range) is reported in Table 1. The majority of adolescents (87.9%) scored below the significant daytime sleepiness cut-off on the Epworth Sleepiness Scale (i.e., <11/24).

**Table 1:** Demographics and Self-Reported Sleep Problems.

Categorical Variables	Number (%)
Females	800 (67.6)
SOL >30 mins	351 (29.6)
NWKS	1.2 (3.14)

Continuous Variables	Mean (SD)
Age (yrs)	15.2 (1.4)
BMI	21.8 (8.6)
Socioeconomic Index (IRSAD)	1013.9 (83.1)
Caffeine; median (IQR)	3.0 (5)
ESS	5.8 (3.9)

BMI  =  Body Mass Index; SOL  =  Sleep Onset Latency; NWKS  =  Number of Wakes; and ESS  =  Epworth Sleepiness Score [28]. Socioeconomic status was estimated using the Index of Relative Socio-economic Advantage and Disadvantage (IRSAD) derived from Socio-Economic Indexes for Areas (SEIFA) Australian census data [29]. Socioeconomic Index data was not available for 7 participants (n = 1177). Caffeine data are the median number of caffeinated drinks consumed in the past week (interquartile range).

### Weekday vs. Weekend Sleep Patterns

Table 2 compares self-reported sleep-wake patterns on weekdays and weekends. Average sleep duration on weekdays (8 hrs 6 mins) was significantly shorter than weekend sleep duration (9 hrs 30 mins), when adolescents extended sleep duration to produce an average catch up sleep of 1 hour 23 mins (*SD* = 01:43). Sleep onset was significantly later on weekends (00:39) than on weekdays (23:50). Adolescents woke significantly later on weekends (10:03) compared with weekdays (07:55), with a mean wake lag of approximately 2 hours 7 mins (*SD* = 01:42). Effect sizes indicated a moderate to large difference in weekday versus weekend sleep onset (*d* = 0.64) and very large effects for weekday/weekend change in sleep duration (*d* = 1.06) and wake time (*d* = 2.63).

**Table 2:** Self-Reported Sleep-Wake Patterns on Weekdays and Weekends.

	Weekday		Weekend			
	M	SD	M	SD	*p*	d
Sleep Duration (hrs)	08:06	01:18	09:30	01:42	<.001	1.06
Sleep Onset Time (24 hr)	23:50	01:18	00:39	01:42	<.001	0.64
Wake Time (24 hr)	07:55	00:48	10:03	01:48	<.001	2.63

d =  Cohen's d. Cohen's D is weekday and weekend mean change divided by SD on weekdays. Mean and SD values are HH:MM.

### Presence and Use of Electronic Devices in Bedrooms at Night

Electronic devices were commonplace in bedrooms with 70.1% (n = 831) of adolescents reporting the presence of two or more devices in their bedroom at night. Eighty per cent (80.2%) reported keeping cellphones in their bedroom at night, 55.2% computers, 41.6% radios, and 32.1% TVs. Figure S1 shows the reported usage of each device in bed during the normal hours of sleep. Almost half (46.8%) reported using their cellphone in bed when they would normally be sleeping on a few nights a week or every, or almost every night. This figure was similarly high (38.5%) for computer use but was lower for use of TVs (23.2%) and radios (15.8%).

### Dose-Response Relationship between Use of Electronic Devices and Sleep Patterns

**Figure 1:**
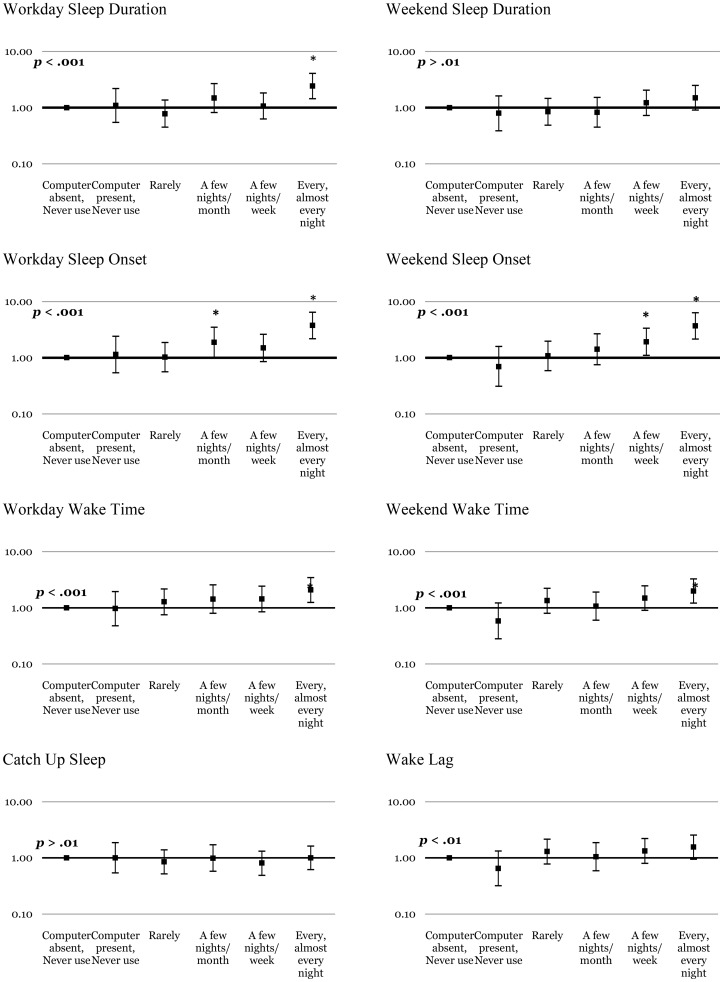
Dose of computer use and the likelihood of problematic sleep. The y axis indicates the odds ratios (bars  = 99% confidence intervals) after controlling for age, gender, socioeconomic status and caffeine use. Stars (*) indicate significantly (*p*<.01) increased likelihood of problematic sleep behaviour for that specific category of use compared with the computer *not* being present (or used) in the sleep environment. P values listed in each panel indicate significance (*α*<.01) of the test for linear trend across increasing doses of computer use.

**Figure 2:**
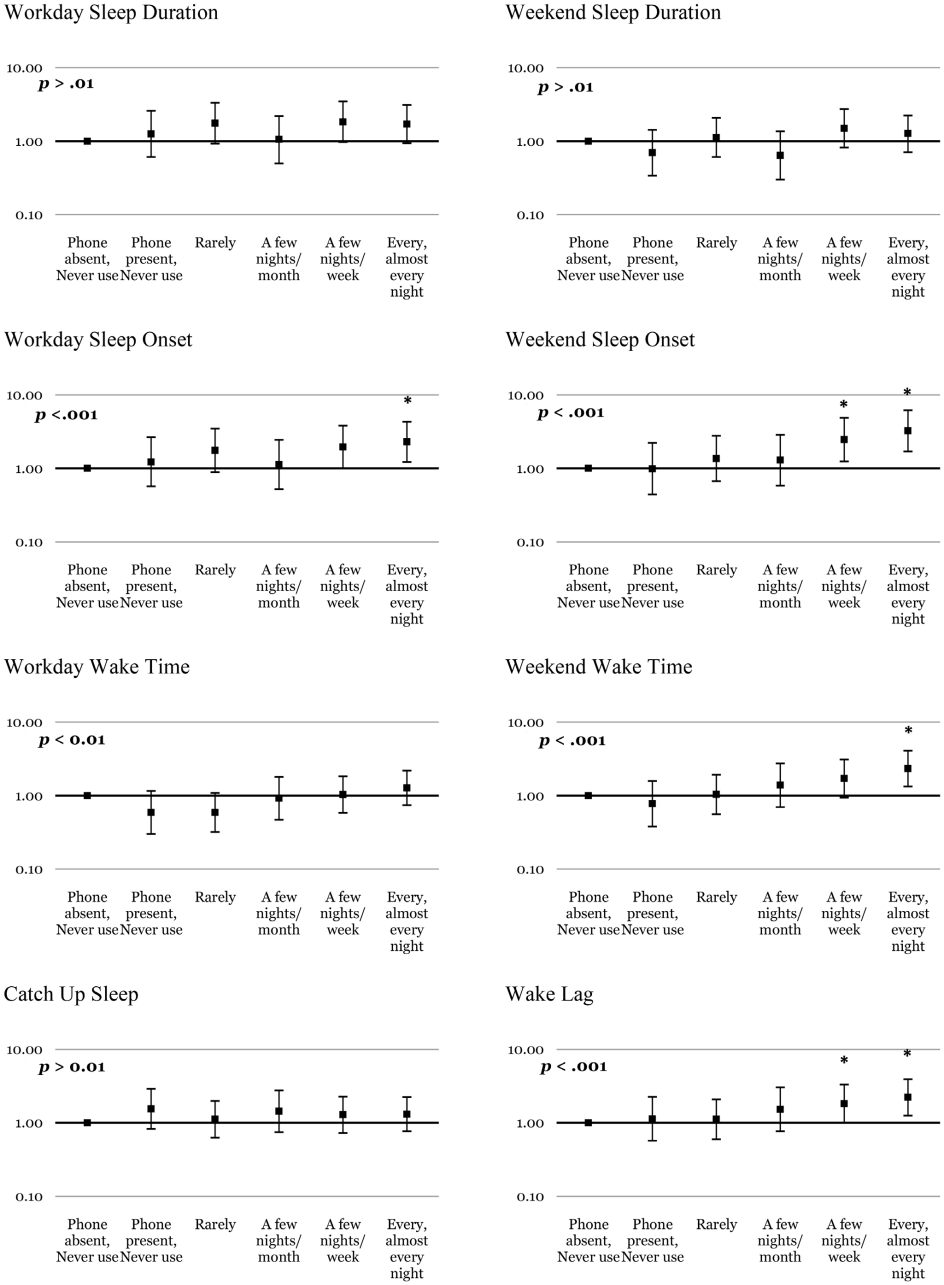
Dose of cellphone use and the likelihood of problematic sleep. The y axis indicates the odds ratios (bars  = 99% confidence intervals) after controlling for age, gender, socioeconomic status and caffeine use. Stars (*) indicate significantly (*p*<.01) increased likelihood of problematic sleep behaviour for that specific category of use compared with the cellphone *not* being present (or used) in the sleep environment. P values listed in each panel indicate significance (*α*<.01) of the test for linear trend across increasing doses of cellphone use.

**Figure 3:**
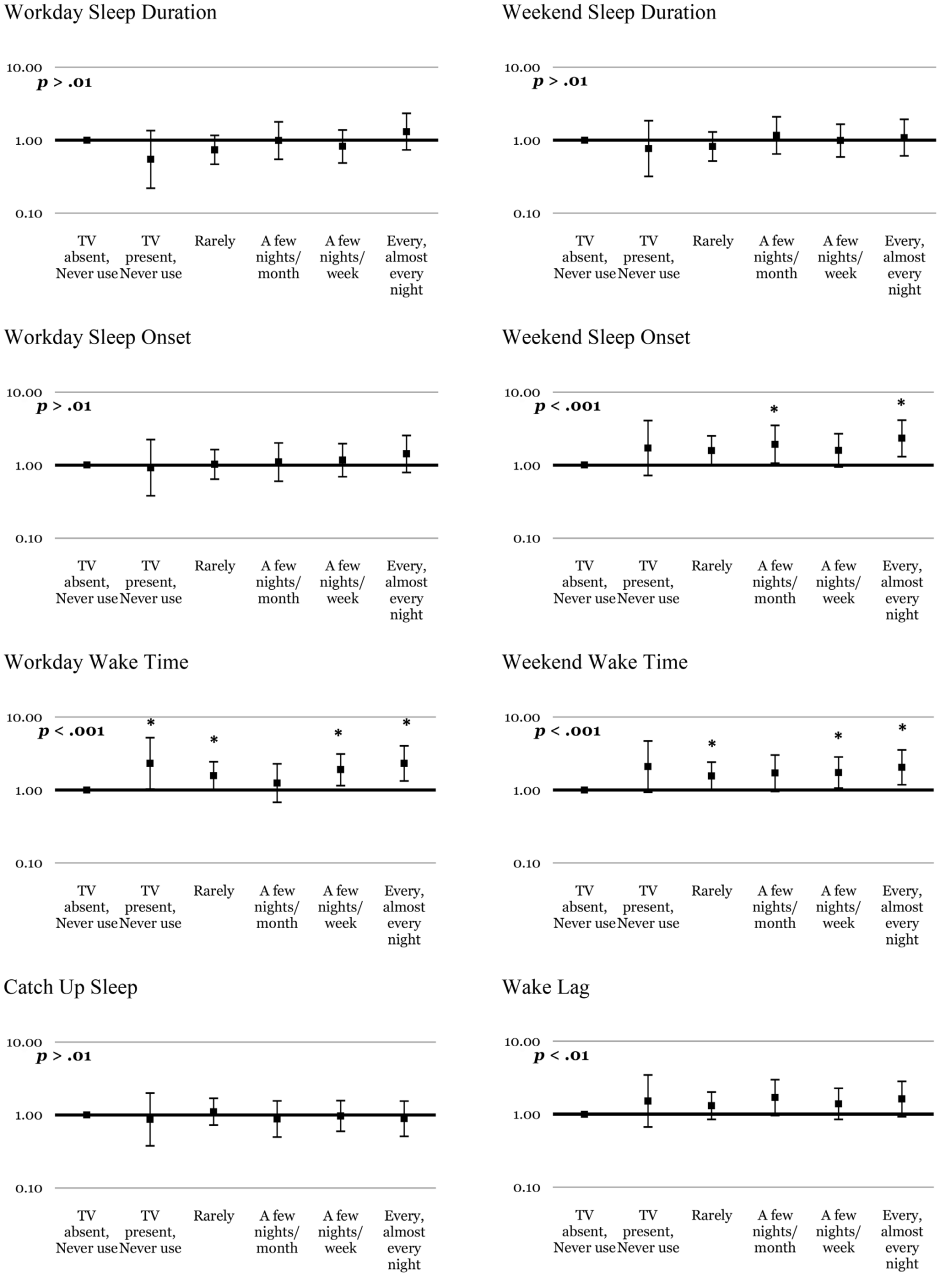
Dose of TV use and the likelihood of problematic sleep. The y axis indicates the odds ratios (bars  = 99% confidence intervals) after controlling for age, gender, socioeconomic status and caffeine use. Stars (*) indicate significantly (*p*<.01) increased likelihood of problematic sleep behaviour for that specific category of use compared with the TV *not* being present (or used) in the sleep environment. P values listed in each panel indicate significance (*α*<.01) of the test for linear trend across increasing doses of TV use.

Multivariate logistic regression models investigating dose-response relationships between the frequency of computer, cellphone, TV and radio use in bed and sleep/wake patterns are summarised in Figures 1–3 & Figure S2. With regard to the overall pattern of dose-response relationships, Figure 1 shows significant linear trends for computer use and short sleep duration on weekdays, later sleep onset and wake times on weekdays and weekends, and greater magnitudes of wake lag. Significant linear trends were found between cellphone use (Figure 2) and later sleep onset and wake times on weekdays and weekends, and greater magnitudes of wake lag. Figure 3 shows significant linear trends for TV use and later sleep onset on weekends, as well as later wake times on weekdays and weekends. No significant linear trends were found for radio use and poor sleep (Figure S2), nor between catch-up sleep and use of any device. The exact numerical values for the odds ratios and associated information in Figures 1–3 and Figure S2 are available in Tables S1–8 in File S1.

## Discussion

We observed dose-response relationships between adolescents' use of computers, cellphones and TVs in bed prior to sleep and delayed sleep/wake patterns, including wake lag (waking later on weekends relative to weekdays). Effects of device use on sleep duration and catch up sleep were less consistent. This pattern of results is consistent with night-time technology use having deleterious effects on both the sleep and circadian systems, potentially impacting on psychological and physical well-being.

Adolescents were heavy users of EDs at night (see Figure S1). Over 70% of adolescents reported two or more devices in their bedroom at night. Almost half (46.8%) reported using their cellphone in bed when they would normally be sleeping a few nights a week or every or almost every night. This figure was similarly high (38.5%) for computer use but was lower for TVs (23.2%) and radios (15.8%). These figures are consistent with studies of American and European [30] adolescents, which have demonstrated heavy use of electronic devices (especially computers and cellphones) at night, with similar effects on sleep.

Computers, TVs and cellphones produced the most consistent dose-dependent effects on adolescent sleep patterns (see Figures 1–3). As predicted, increasing night-time use of computers was associated with increasingly short sleep duration on weekdays, and later sleep onset and wake times on weekdays and weekends, as well as greater than average wake lag. TV use was also associated with later sleep onset on weekends, as well as progressively later waking on weekdays and weekends, and greater than average wake lag. Use of cellphones was associated with delayed sleep onset and wake times on weekdays and weekends, as well as greater than average wake lag.

Importantly, dose-response relationships between computer, TV and cellphone use and sleep were observed over and above the general tendency for sleep/wake irregularities in adolescents. On average, adolescents in our sample fell asleep one hour later and woke two hours later on weekends relative to weekdays. Sleep duration on weekdays fell on the borderline of insufficient/sufficient sleep for this age group at 8.1 hours, but on weekends averaged 9.5 hours. These sleep patterns are consistent with normative data for this age group [26], but the significant dose-response relationships observed raise concerns of an additive risk of poor sleep with use of EDs. Delayed sleep onset and wake patterns linked with ED use are important because of demonstrated links with a range of adverse outcomes including daytime sleepiness [10], impaired academic performance [11], and increased risk taking behaviour, stimulant consumption and depressed mood [7].

Unlike computers, cellphones and TVs, use of radios in bed did not show dose-response relationships with sleep (See Figure S2). Usage rates for radios were similar to TVs, suggesting that the study was not underpowered to detect dose-response effects for radios and sleep. Stronger effects for computers, TVs and cellphones compared with radios likely reflect differences in the nature of these devices and their mechanism of action. First, unlike radios, computers, cellphones and TVs emit light, known to suppress melatonin and delay sleep/wake patterns [31]. This study surveyed use of devices in bed, potentially emphasizing either large-screen devices that may be comfortably viewed from bed, or the use of smaller hand-held devices that tend to be used at a closer viewing distance producing greater light intensity [31]. Second, computers and cellphones are interactive devices and their use in bed may be more arousing or alerting than listening to the radio in bed, which may be considered to be more passive. Frequent use of such interactive devices in bed may disrupt sleep by creating a learned-association between the bed and wakefulness. Hence the pattern of results may demonstrate the power of light-emitting interactive devices to delay sleep/wake patterns, either via circadian systems, or via conditioned arousal, or through some combination. There is need for future research to investigate the specific mechanism(s) by which devices disrupt sleep.

Linear trends (Figures 1–3) support dose-dependent relationships between increasing use of computers, TVs or cellphones in bed and progressively later sleep onset and wake times, and wake lag. Notably, odds ratios for sleep problems increased significantly when computers, TVs and cellphones were used a few nights per week or more, suggesting that frequent use in bed is required to produce effects on sleep. Less frequent use (never to a few nights per month) of these devices in bed was generally not associated with significant odds of problematic sleep suggesting that infrequent use does not significantly increase the risk of poor sleep. Such ‘real world’ advice is important considering that adolescents are heavy users of EDs at night [21] and are unlikely to abstain completely from their use.

Our results also support the intuitive assumption that it is the use of electronic devices, rather than their presence in the bedroom that is important for sleep. Sleep hygiene advice generally recommends the removal of EDs from bedrooms. However, at least in this sample, there was only one instance in which an unused bedroom device was associated with increased risk sleep problems. Keeping a TV in the bedroom but not using it predicted later waking on weekdays, but otherwise there were no effects for unused devices on any sleep/wake outcome. Overall, results suggest that adolescents unable to limit their usage of computers, TVs and cellphones to less than a few nights per week may be at increased risk of sleep problems.

The current study involved an open online self-reported survey about technology use. The sample is self-selected and includes cross-sectional data only and may not have captured typical use by the average adolescent. It is not known whether the documented dose-response relationships between ED use and sleep would be observed in a population-representative sample or whether the association is due to reverse-causation (technology use could be a symptom not a cause of poor sleep [32]) or whether we would have observed the same patterns if we had phrased the exposure measure question differently. This study surveyed devices that were widely used in Australian popular culture in 2010/2011. Invariably research in this field is outpaced by the proliferation of newer and more advanced devices and our results would be improved by including a broader range of EDs including handheld music devices (e.g. iPod, MP3 player), gaming consoles, and 3^rd^ generation devices (internet interactive devices or smart phones). Our results suggest that frequent use of light-emitting interactive devices in bed delays sleep and wake timing in adolescents, either through the circadian system (i.e., melatonin suppression), conditioning the bed as a place of arousal, or some combination. Thus, future research must ask about a broad range of devices, but also be specific about how and why they influence sleep. Night-time ED use may be an important target for intervention and prevention of ill-health in young people. To aid the development of evidence-based guidelines for safe use of EDs it would be useful to see clinical trials of ED restriction to treat or prevent sleep problems in high users of EDs.

Electronic devices (EDs) are commonly present and frequently used in the bedrooms of adolescents. This is the first study to observe dose-dependent associations between the use of EDs in bed (especially computers, TVs and cellphones) and effects on sleep and circadian behaviours, including relatively later sleep onset and wake times and wake lag (later waking on weekends vs. weekdays). These dose-dependent associations were observed over and above the general tendency for sleep/wake irregularities in adolescents and were strongest for light-emitting interactive devices such as computers, TVs and cellphones. The mere presence of devices in the bedroom was not associated with poor sleep. The overall pattern of results suggests that use of devices in bed a few nights a week or more delays sleep onset and wake times in adolescents, whilst less frequent use was not associated with deleterious sleep schedules. The latter is important for developing feasible guidelines for night-time ED use and healthy sleep in adolescents, particularly in light of their current heavy use and the potential flow-on effects for physical and mental well-being.

## Supporting Information

Figure S1 Percentage of adolescents reporting using Computers, Cell phones, TVs and Radios in bed during the normal hours of sleep.
(TIF)

Figure S2 Dose of radio use and the likelihood of problematic sleep. The y axis indicates the odds ratios (bars  = 99% confidence intervals) after controlling for age, gender, socioeconomic status and caffeine use. Stars (*) indicate significantly (*p*<.01) increased likelihood of problematic sleep behaviour for that specific category of use compared with the Radio *not* being present (or used) in the sleep environment. P values listed in each panel indicate significance (*α*<.01) of the test for linear trend across increasing doses of radio use.
(TIF)

File S1 Contains Table S1–S8.
(DOC)
