# Supplementary figures and images for: Adolescent Sleep Patterns and Night-Time Technology Use: Results of the Australian Broadcasting Corporation's Big Sleep Survey
Source: PLoS One. 2014 Nov 12;9(11):e111700. doi: 10.1371/journal.pone.0111700 (PMC4229101; doi:10.1371/journal.pone.0111700)

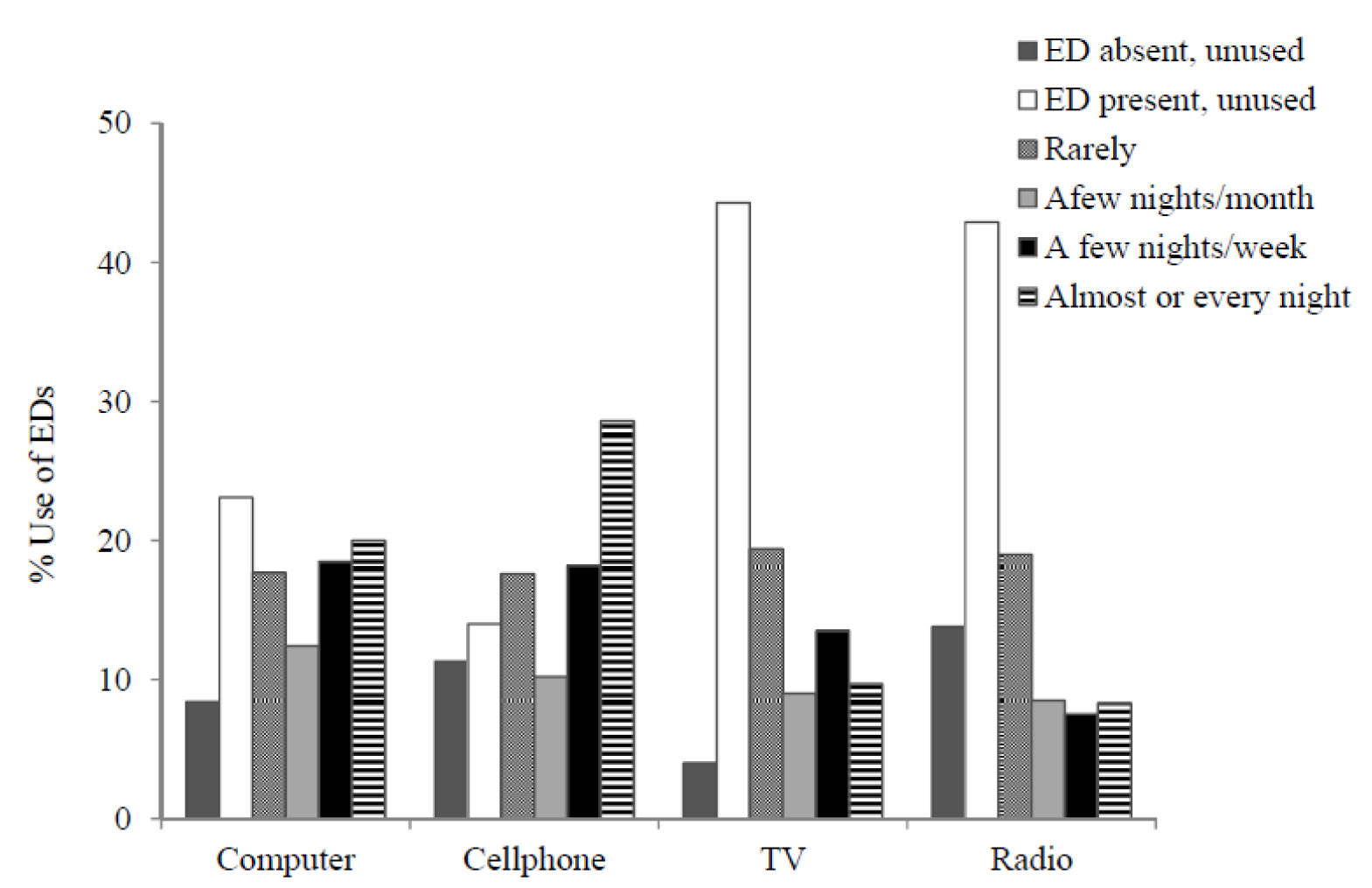

Supplement: Figure S1 — Percentage of adolescents reporting using Computers, Cell phones, TVs and Radios in bed during the normal hours of sleep. (TIF) [file pone.0111700.s001.tif]

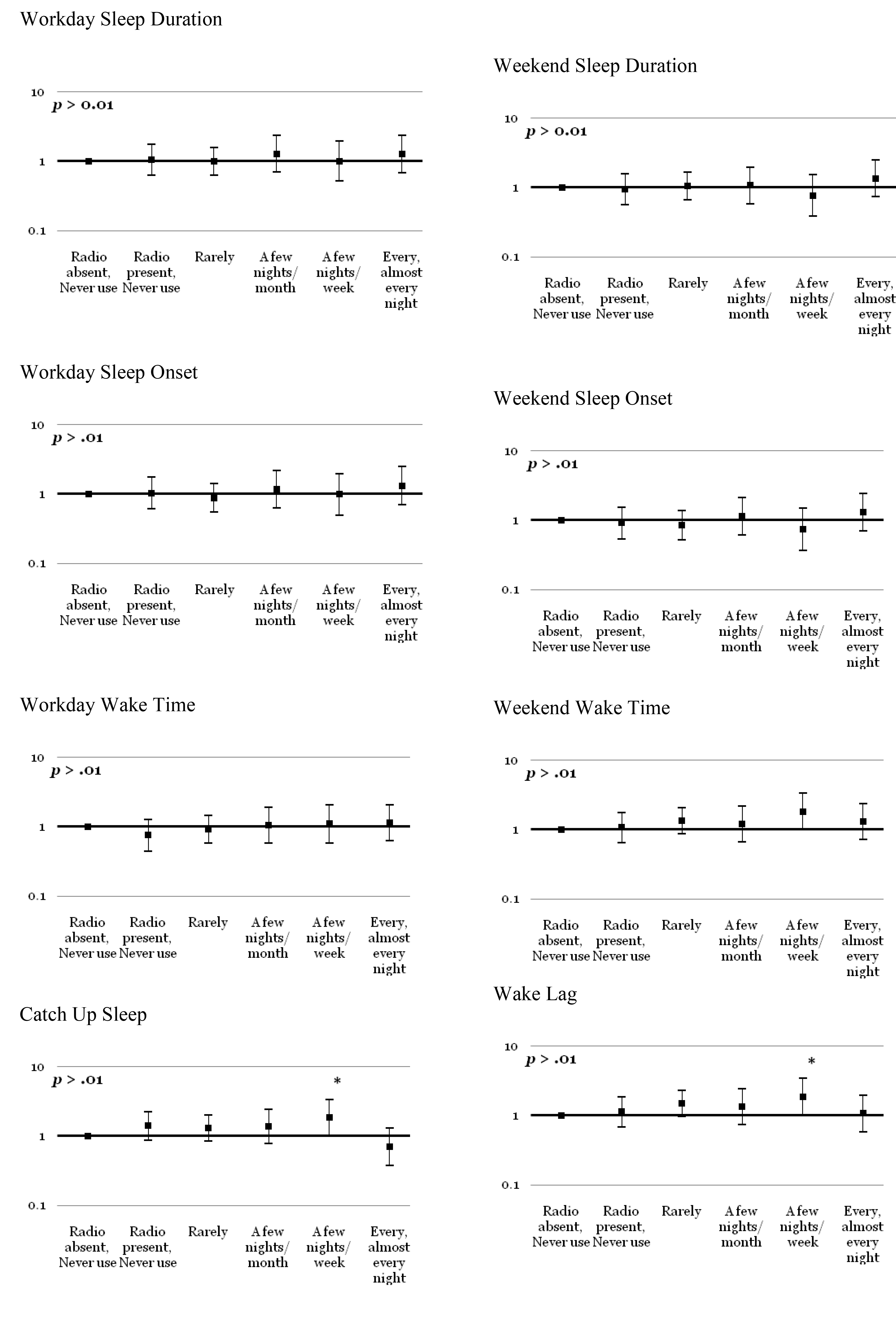

Supplement: Figure S2 — Dose of radio use and the likelihood of problematic sleep. The y axis indicates the odds ratios (bars = 99% confidence intervals) after controlling for age, gender, socioeconomic status and caffeine use. Stars (*) indicate significantly (p<.01) increased likelihood of problematic sleep behaviour for that specific category of use compared with the Radio not being present (or used) in the sleep environment. P values listed in each panel indicate significance (α<.01) of the test for linear trend across increasing doses of radio use. (TIF) [file pone.0111700.s002.tif]
