# Supplementary material for: Adolescent Sleep Patterns and Night-Time Technology Use: Results of the Australian Broadcasting Corporation's Big Sleep Survey
Source: PLoS One. 2014 Nov 12;9(11):e111700. doi: 10.1371/journal.pone.0111700 (PMC4229101; doi:10.1371/journal.pone.0111700)
Supplement: File S1 — Contains Table S1–S8. (DOC) [file pone.0111700.s003.doc]

Supplementary Table 1. Dose Dependent Associations between Electronic Device use and Weekday Sleep Duration including raw frequencies, univariate and multivariate associations.

|  | Quintiles N (%) | Device Absent, Never Use | Device Present, Never Use | Rarely | A Few nights/month | A Few nights/ week | Every or almost every night | P for Trend |
| --- | --- | --- | --- | --- | --- | --- | --- | --- |
| Computer | Normal 3 Quintiles 727 (61.4) | 195 (71.4) | 65 (65.7) | 151 (72.2) | 82 (55.8) | 134 (61.2) | 100 (42.2) |  |
|  | Abnormal 2 Quintiles 457 (38.6) | 78 (28.6) | 34 (34.3) | 58 (27.8) | 65 (44.2) | 85 (38.8) | 137 (57.8) |  |
|  | Multivariate OR (99 CI) | 1.0 | 1.10 (0.55, 2.20) | 0.78 (0.45, 1.37) | 1.49 (0.82, 2.69) | 1.07 (0.63, 1.83) | 2.43 (1.45, 4.08) | <.001 |
|  | Univariate OR (99 CI) | 1.0 | 1.31 (0.69, 2.49) | 0.96 (0.57, 1.63) | 1.99 (1.14, 3.43) | 1.59 (0.97, 2.61) | 3.43 (2.11, 5.56) | <.001 |
| Mobile | Normal 3 Quintiles 727 (61.4) | 127 (76.5) | 92 (68.7) | 124 (59.6) | 81 (66.9) | 123 (56.9) | 180 (53.1) |  |
|  | Abnormal 2 Quintiles 457 (38.6) | 39 (23.5) | 42 (31.3) | 84 (40.4) | 40 (33.1) | 93 (43.1) | 159 (46.9) |  |
|  | Multivariate OR (99 CI) | 1.0 | 1.26 (0.61, 2.61) | 1.76 (0.93, 3.35) | 1.06 (0.50, 2.20) | 1.83 (0.97, 3.47) | 1.71 (0.94, 3.12) | .021 |
|  | Univariate OR (99 CI) | 1.0 | 1.49 (0.76, 2.91) | 2.21 (1.22, 4.00) | 1.61 (0.81, 3.19) | 2.46 (1.37, 4.44) | 2.88 (1.66, 4.98) | <.001 |
| TV | Normal 3 Quintiles 727 (61.4) | 327 (62.3) | 33 (70.2) | 149 (64.8) | 59 (55.1) | 99 (61.9) | 60 (52.2) |  |
|  | Abnormal 2 Quintiles 457 (38.6) | 198 (37.7) | 14 (29.8) | 81 (35.2) | 48 (44.9) | 61 (38.1) | 55 (47.8) |  |
|  | Multivariate OR (99 CI) | 1.0 | 0.55 (0.22, 1.35) | 0.74 (0.47, 1.17) | 0.99 (0.55, 1.78) | 0.83 (0.49, 1.38) | 1.31 (0.74, 2.33) | .726 |
|  | Univariate OR (99 CI) | 1.0 | 0.70 (0.30, 1.65) | 0.90 (0.59,1.37) | 1.34 (0.77, 2.33) | 1.02 (0.63, 1.64) | 1.51 (0.89, 2.58) | .095 |
| Radio | Normal 3 Quintiles 727 (61.4) | 326 (64.2) | 97 (59.5) | 138 (61.3) | 54 (53.5) | 57 (64.0) | 55 (56.1) |  |
|  | Abnormal 2 Quintiles 457 (38.6) | 182 (35.8) | 66 (40.5) | 87 (38.7) | 47 (46.5) | 32 (36.0) | 43 (43.9) |  |
|  | Multivariate OR (99 CI) | 1.0 | 1.05 (0.63, 1.75) | 1.01 (0.64, 1.59) | 1.28 (0.70, 2.35) | 1.00 (0.52, 1.94) | 1.27 (0.69, 2.35) | .341 |
|  | Univariate OR (99 CI) | 1.0 | 1.22 (0.76, 1.96) | 1.13 (0.74, 1.73) | 1.56 (0.89, 2.75) | 1.01 (0.54, 1.87) | 1.40 (0.79, 2.49) | .113 |

Supplementary Table 2: Dose Dependent Associations between Electronic Device use and Weekday Sleep Onset including raw frequencies, univariate and multivariate associations.

|  | Quintiles N (%) | Device Absent, Never Use | Device Present, Never Use | Rarely | A Few nights/month | A Few nights/ week | Every or almost every night | P for Trend |
| --- | --- | --- | --- | --- | --- | --- | --- | --- |
| Computer | Normal 3 Quintiles 770 (65) | 72 (72.7) | 214 (78.4) | 157 (75.1) | 88 (59.9) | 139 (63.5) | 100 (42.2) |  |
|  | Abnormal 2 Quintiles 414 (35) | 27 (27.3) | 59 (21.6) | 52 (24.9) | 59 (40.1) | 80 (36.5) | 137 (57.8) |  |
|  | Multivariate OR (99 CI) | 1.0 | 1.14 (0.54, 2.41) | 1.02 (0.56, 1.86) | 1.88 (1.01, 3.49) | 1.49 (0.85, 2.61) | 3.75 (2.17, 6.46) | <.001 |
|  | Univariate OR (99 CI) | 1.0 | 1.36 (0.68, 2.72) | 1.20 (0.69, 2.10) | 2.43 (1.37, 4.32) | 2.09 (1.24, 3.52) | 4.97 (2.99, 8.26) | <.001 |
| Mobile | Normal 3 Quintiles 770 (65.0) | 99 (73.9) | 134 (80.7) | 137 (65.9) | 86 (71.1) | 134 (62.0) | 180 (53.1) |  |
|  | Abnormal 2 Quintiles 414 (35.0) | 35 (26.1) | 32 (19.3) | 71 (34.1) | 35 (28.9) | 82 (38.0) | 159 (46.9) |  |
|  | Multivariate OR (99 CI) | 1.0 | 1.22 (0.57, 2.65) | 1.75 (0.89, 3.46) | 1.12 (0.52, 2.44) | 1.95 (1.00, 3.81) | 2.29 (1.22, 4.30) | <.001 |
|  | Univariate OR (99 CI) | 1.0 | 1.48 (0.72, 3.03) | 2.17 (1.15, 4.08) | 1.70 (0.83, 3.51) | 2.56 (1.38, 4.78) | 3.70 (2.07, 6.60) | <.001 |
| TV | Normal 3 Quintiles 770 (65.0) | 31 (66.0) | 362 (69.0) | 149 (64.8) | 64 (59.8) | 98 (61.2) | 66 (57.4) |  |
|  | Abnormal 2 Quintiles 414 (35.0) | 16 (34.0) | 163 (31.0) | 81 (35.2) | 43 (40.2) | 62 (38.8) | 49 (42.6) |  |
|  | Multivariate OR (99 CI) | 1.0 | 0.91 (0.38, 2.23) | 1.02 (0.64, 1.63) | 1.10 (0.60, 2.01) | 1.17 (0.69, 1.97) | 1.42 (0.79, 2.54) | .133 |
|  | Univariate OR (99 CI) | 1.0 | 1.15 (0.50, 2.63) | 1.21 (0.79, 1.86) | 1.49 (0.85, 2.62) | 1.41 (0.87, 2.28) | 1.65 (0.96, 2.84) | .004 |
| Radio | Normal 3 Quintiles 770 (65.0) | 102 (62.6) | 341 (67.1) | 151 (67.1) | 59 (58.4) | 60 (67.4) | 57 (58.2) |  |
|  | Abnormal 2 Quintiles 414 (35.0) | 61 (37.4) | 167 (32.9) | 74 (32.9) | 42 (41.6) | 29 (32.6) | 41 (41.8) |  |
|  | Multivariate OR (99 CI) | 1.0 | 1.04 (0.62, 1.75) | 0.88 (0.55, 1.41) | 1.19 (0.64, 2.21) | 0.99 (0.50, 1.94) | 1.32 (0.71, 2.47) | .408 |
|  | Univariate OR (99 CI) | 1.0 | 1.22 (0.75, 1.98) | 1.00 (0.65, 1.55) | 1.45 (0.82, 2.58) | 0.99 (0.53, 1.86) | 1.47 (0.82, 2.63) | .141 |

Supplementary Table 3. Dose Dependent Associations between Electronic Device use and Weekday Wake Time including raw frequencies, univariate and multivariate associations.

|  | Quintiles N (%) | Device Absent, Never Use | Device Present, Never Use | Rarely | A Few nights/month | A Few nights/ week | Every or almost every night | P for Trend |
| --- | --- | --- | --- | --- | --- | --- | --- | --- |
| Computer | Normal 3 Quintiles 798 (67.4) | 74 (74.7) | 202 (74.0) | 144 (68.9) | 97 (66.0) | 145 (66.2) | 136 (57.4) |  |
|  | Abnormal 2 Quintiles 386 (32.6) | 25 (25.3) | 71 (26.0) | 65 (31.1) | 50 (34.0) | 74 (33.8) | 101 (42.6) |  |
|  | Multivariate OR (99 CI) | 1.0 | 0.97 (0.48, 1.95) | 1.28 (0.75, 2.17) | 1.43 (0.80, 2.56) | 1.44 (0.85, 2.43) | 2.08 (1.25, 3.44) | <.001 |
|  | Univariate OR (99 CI) | 1.0 | 0.96 (0.48, 1.92) | 1.28 (0.76, 2.17) | 1.47 (0.83, 2.60) | 1.45 (0.87, 2.42) | 2.11 (1.29, 3.45) | <.001 |
| Mobile | Normal 3 Quintiles 798 (67.4) | 103 (76.9) | 110 (66.3) | 160 (76.9) | 81 (66.9) | 141 (65.3) | 203 (59.9) |  |
|  | Abnormal 2 Quintiles 386 (32.6) | 31 (23.1) | 56 (33.7) | 48 (23.1) | 40 (33.1) | 75 (34.7) | 136 (40.1) |  |
|  | Multivariate OR (99 CI) | 1.0 | 0.59 (0.30, 1.16) | 0.59 (0.32, 1.09) | 0.92 (0.47, 1.79) | 1.03 (0.58, 1.83) | 1.27 (0.74, 2.18) | .004 |
|  | Univariate OR (99 CI) | 1.0 | 0.59 (0.30, 1.16) | 0.59 (0.32, 1.07) | 0.97 (0.51, 1.86) | 1.05 (0.60, 1.83) | 1.32 (0.79, 2.19) | .001 |
| TV | Normal 3 Quintiles 798 (67.4) | 26 (55.3) | 391 (74.5) | 148 (64.3) | 74 (69.2) | 96 (60.0) | 63 (54.8) |  |
|  | Abnormal 2 Quintiles 386 (32.6) | 21 (44.7) | 134 (25.5) | 82 (35.7) | 33 (30.8) | 64 (40.0) | 52 (45.2) |  |
|  | Multivariate OR (99 CI) | 1.0 | 2.31 (1.03, 5.19) | 1.57 (1.01, 2.45) | 1.25 (0.68, 2.28) | 1.90 (1.15, 3.12) | 2.31 (1.33, 4.02) | <.001 |
|  | Univariate OR (99 CI) | 1.0 | 2.36 (1.06, 5.24) | 1.62 (1.04, 2.51) | 1.30 (0.72, 2.37) | 1.95 (1.19, 3.17) | 2.41 (1.39, 4.16) | <.001 |
| Radio | Normal 3 Quintiles 798 (67.4) | 118 (72.4) | 340 (66.9) | 154 (68.4) | 66 (65.3) | 58 (65.2) | 62 (63.3) |  |
|  | Abnormal 2 Quintiles 386 (32.6) | 45 (27.6) | 168 (33.1) | 71 (31.6) | 35 (34.7) | 31 (34.8) | 36 (36.7) |  |
|  | Multivariate OR (99 CI) | 1.0 | 0.76 (0.45, 1.27) | 0.92 (0.59, 1.44) | 1.06 (0.59, 1.93) | 1.10 (0.59, 2.06) | 1.14 (0.63, 2.08) | .452 |
|  | Univariate OR (99 CI) | 1.0 | 0.77 (0.46, 1.29) | 0.93 (0.60, 1.45) | 1.07 (0.59, 1.94) | 1.08 (0.58, 2.02) | 1.18 (0.65, 2.12) | .418 |

Supplementary Table 4. Dose Dependent Associations between Electronic Device use and Weekend Sleep Duration including raw frequencies, univariate and multivariate associations.

|  | Quintiles N (%) | Device Absent, Never Use | Device Present, Never Use | Rarely | A Few nights/month | A Few nights/ week | Every or almost every night | P for Trend |
| --- | --- | --- | --- | --- | --- | --- | --- | --- |
| Computer | Normal 3 Quintiles 811 (68.5) | 74 (74.7) | 198 (72.5) | 154 (73.7) | 106 (72.1) | 140 (63.9) | 139 (58.6) |  |
|  | Abnormal 2 Quintiles 373 (31.5) | 25 (25.3) | 75 (27.5) | 55 (26.3) | 41 (27.9) | 79 (36.1) | 98 (41.4) |  |
|  | Multivariate OR (99 CI) | 1.0 | 0.80 (0.39, 1.62) | 0.85 (0.49, 1.47) | 0.83 (0.45, 1.53) | 1.22 (0.73, 2.06) | 1.50 (0.91, 2.50) | .011 |
|  | Univariate OR (99 CI) | 1.0 | 0.89 (0.45, 1.78) | 0.94 (0.55, 1.61) | 1.02 (0.57, 1.84) | 1.49 (0.90, 2.46) | 1.86 (1.14, 3.03) | <.001 |
| Mobile | Normal 3 Quintiles 811 (68.5) | 104 (77.6) | 122 (73.5) | 143 (68.8) | 93 (76.9) | 134 (62.0) | 215 (63.4) |  |
|  | Abnormal 2 Quintiles 373 (31.5) | 30 (22.4) | 44 (26.5) | 65 (31.2) | 28 (23.1) | 82 (38.0) | 124 (36.6) |  |
|  | Multivariate OR (99 CI) | 1.0 | 0.70 (0.34, 1.43) | 1.12 (0.61, 2.08) | 0.64 (0.30, 1.36) | 1.50 (0.82, 2.75) | 1.27 (0.71, 2.25) | .022 |
|  | Univariate OR (99 CI) | 1.0 | 0.80 (0.40, 1.61) | 1.26 (0.70, 2.28) | 0.84 (0.41, 1.71) | 1.70 (0.95, 3.03) | 1.60 (0.93, 2.74) | .001 |
| TV | Normal 3 Quintiles 811 (68.5) | 33 (70.2) | 365 (69.5) | 164 (71.3) | 67 (62.6) | 107 (66.9) | 75 (65.2) |  |
|  | Abnormal 2 Quintiles 373 (31.5) | 14 (29.8) | 160 (30.5) | 66 (28.7) | 40 (37.4) | 53 (33.1) | 40 (34.8) |  |
|  | Multivariate OR (99 CI) | 1.0 | 0.77 (0.32, 1.86) | 0.82 (0.52, 1.30) | 1.17 (0.65, 2.10) | 0.99 (0.59, 1.66) | 1.08 (0.61, 1.93) | .704 |
|  | Univariate OR (99 CI) | 1.0 | 0.97 (0.41, 2.28) | 0.92 (0.59, 1.44) | 1.36 (0.77, 2.41) | 1.13 (0.69, 1.86) | 1.22 (0.70, 2.13) | .232 |
| Radio | Normal 3 Quintiles 811 (68.5) | 112 (68.7) | 353 (69.5) | 151 (67.1) | 67 (66.3) | 67 (75.3) | 61 (62.2) |  |
|  | Abnormal 2 Quintiles 373 (31.5) | 51 (31.3) | 155 (30.5) | 74 (32.9) | 34 (33.7) | 22 (24.7) | 37 (37.8) |  |
|  | Multivariate OR (99 CI) | 1.0 | 0.95 (0.57, 1.58) | 1.06 (0.67, 1.66) | 1.07 (0.58, 1.98) | 0.77 (0.39, 1.55) | 1.35 (0.74, 2.48) | .538 |
|  | Univariate OR (99 CI) | 1.0 | 1.04 (0.63, 1.71) | 1.12 (0.72, 1.74) | 1.16 (0.64, 2.10) | 0.75 (0.38, 1.48) | 1.38 (0.77, 2.50) | .473 |

Supplementary Table 5. Dose Dependent Associations between Electronic Device use and Weekend Sleep Onset including raw frequencies, univariate and multivariate associations.

|  | Quintiles N (%) | Device Absent, Never Use | Device Present, Never Use | Rarely | A Few nights/month | A Few nights/ week | Every or almost every night | P for Trend |
| --- | --- | --- | --- | --- | --- | --- | --- | --- |
| Computer | Normal 3 Quintiles 803 (67.8) | 82 (82.8) | 219 (80.2) | 160 (76.6) | 100 (68.0) | 134 (61.2) | 108 (45.6) |  |
|  | Abnormal 2 Quintiles 381 (32.2) | 17 (17.2) | 54 (19.8) | 49 (23.4) | 47 (32.0) | 85 (38.8) | 129 (54.4) |  |
|  | Multivariate OR (99 CI) | 1.0 | 0.69 (0.31, 1.58) | 1.08 (0.59, 1.96) | 1.41 (0.75, 2.66) | 1.92 (1.10, 3.35) | 3.68 (2.14, 6.32) | <.001 |
|  | Univariate OR (99 CI) | 1.0 | 0.84 (0.38, 1.85) | 1.24 (0.70, 2.21) | 1.91 (1.05, 3.48) | 2.57 (1.51, 4.37) | 4.84 (2.89, 8.11) | <.001 |
| Mobile | Normal 3 Quintiles 803 (67.8) | 108 (80.6) | 139 (83.7) | 156 (75.0) | 88 (72.7) | 135 (62.5) | 177 (52.2) |  |
|  | Abnormal 2 Quintiles 381 (32.2) | 26 (19.4) | 27 (16.3) | 52 (25.0) | 33 (27.3) | 81 (37.5) | 162 (47.8) |  |
|  | Multivariate OR (99 CI) | 1.0 | 0.98 (0.44, 2.22) | 1.36 (0.67, 2.77) | 1.29 (0.58, 2.85) | 2.45 (1.24, 4.84) | 3.24 (1.70, 6.19) | <.001 |
|  | Univariate OR (99 CI) | 1.0 | 1.24 (0.57, 2.71) | 1.72 (0.87, 3.39) | 1.93 (0.91, 4.12) | 3.09 (1.61, 5.93) | 4.71 (2.56, 8.67) | <.001 |
| TV | Normal 3 Quintiles 803 (67.8) | 29 (61.7) | 399 (76.0) | 148 (64.3) | 61 (57.0) | 102 (63.8) | 64 (55.7) |  |
|  | Abnormal 2 Quintiles 381 (32.2) | 18 (38.3) | 126 (24.0) | 82 (35.7) | 46 (43.0) | 58 (36.2) | 51 (44.3) |  |
|  | Multivariate OR (99 CI) | 1.0 | 1.71 (0.72, 4.07) | 1.57 (0.99, 2.51) | 1.92 (1.06, 3.48) | 1.58 (0.94, 2.68) | 2.32 (1.30, 4.14) | <.001 |
|  | Univariate OR (99 CI) | 1.0 | 1.97 (0.87, 4.45) | 1.76 (1.13, 2.73) | 2.39 (1.35, 4.21) | 1.80 (1.09, 2.97) | 2.52 (1.46, 4.38) | <.001 |
| Radio | Normal 3 Quintiles 803 (67.8) | 110 (67.5) | 348 (68.5) | 158 (70.2) | 62 (61.4) | 66 (74.2) | 59 (60.2) |  |
|  | Abnormal 2 Quintiles 381 (32.2) | 53 (32.5) | 160 (31.5) | 67 (29.8) | 39 (38.6) | 23 (25.8) | 39 (39.8) |  |
|  | Multivariate OR (99 CI) | 1.0 | 0.91 (0.54, 1.54) | 0.85 (0.53, 1.37) | 1.15 (0.62, 2.14) | 0.75 (0.37, 1.51) | 1.30 (0.70, 2.42) | .700 |
|  | Univariate OR (99 CI) | 1.0 | 1.05 (0.64, 1.72) | 0.92 (0.59, 1.45) | 1.37 (0.77, 2.45) | 0.76 (0.39, 1.48) | 1.44 (0.80, 2.58) | .366 |

Supplementary Table 6. Dose Dependent Associations between Electronic Device use and Weekend Wake Time including raw frequencies, univariate and multivariate associations.

|  | Quintiles N (%) | Device Absent, Never Use | Device Present, Never Use | Rarely | A Few nights/month | A Few nights/ week | Every or almost every night | P for Trend |
| --- | --- | --- | --- | --- | --- | --- | --- | --- |
| Computer | Normal 3 Quintiles 754 (63.7) | 78 (78.8) | 192 (70.3) | 132 (63.2) | 99 (67.3) | 130 (59.4) | 123 (51.9) |  |
|  | Abnormal 2 Quintiles 430 (36.3) | 21 (21.2) | 81 (29.7) | 77 (36.8) | 48 (32.7) | 89 (40.6) | 114 (48.1) |  |
|  | Multivariate OR (99 CI) | 1.0 | 0.58 (0.28, 1.22) | 1.34 (0.80, 2.23) | 1.07 (0.60, 1.91) | 1.49 (0.90, 2.47) | 1.99 (1.21, 3.26) | <.001 |
|  | Univariate OR (99 CI) | 1.0 | 0.64 (0.31, 1.31) | 1.38 (0.84, 2.29) | 1.15 (0.65, 2.03) | 1.62 (0.99, 2.65) | 2.20 (1.36, 3.54) | <.001 |
| Mobile | Normal 3 Quintiles 754 (63.7) | 103 (76.9) | 122 (73.5) | 149 (71.6) | 78 (64.5) | 130 (60.2) | 172 (50.7) |  |
|  | Abnormal 2 Quintiles 430 (36.3) | 31 (23.1) | 44 (26.5) | 59 (28.4) | 43 (35.5) | 86 (39.8) | 167 (49.3) |  |
|  | Multivariate OR (99 CI) | 1.0 | 0.78 (0.38, 1.58) | 1.04 (0.56, 1.92) | 1.39 (0.70, 2.75) | 1.71 (0.94, 3.09) | 2.33 (1.33, 4.08) | <.001 |
|  | Univariate OR (99 CI) | 1.0 | 0.84 (0.42, 1.67) | 1.10 (0.60, 2.00) | 1.53 (0.79, 2.98) | 1.83 (1.03, 3.27) | 2.69 (1.58, 4.59) | <.001 |
| TV | Normal 3 Quintiles 754 (63.7) | 25 (53.2) | 376 (71.6) | 139 (60.4) | 61 (57.0) | 92 (57.5) | 61 (53.0) |  |
|  | Abnormal 2 Quintiles 430 (36.3) | 22 (46.8) | 149 (28.4) | 91 (39.6) | 46 (43.0) | 68 (42.5) | 54 (47.0) |  |
|  | Multivariate OR (99 CI) | 1.0 | 2.10 (0.93, 4.71) | 1.56 (1.01, 2.41) | 1.71 (0.96, 3.02) | 1.74 (1.06, 2.84) | 2.04 (1.18, 3.55) | <.001 |
|  | Univariate OR (99 CI) | 1.0 | 2.22 (1.01, 4.91) | 1.65 (1.08, 2.53) | 1.90 (1.09, 3.34) | 1.87 (1.15, 3.02) | 2.23 (1.30, 3.84) | <.001 |
| Radio | Normal 3 Quintiles 754 (63.7) | 106 (65.0) | 344 (67.7) | 135 (60.0) | 63 (62.4) | 48 (53.9) | 58 (59.2) |  |
|  | Abnormal 2 Quintiles 430 (36.3) | 57 (35.0) | 164 (32.3) | 90 (40.0) | 38 (37.6) | 41 (46.1) | 40 (40.8) |  |
|  | Multivariate OR (99 CI) | 1.0 | 1.07 (0.65, 1.75) | 1.35 (0.87, 2.08) | 1.20 (0.66, 2.17) | 1.83 (0.99, 3.38) | 1.30 (0.72, 2.36) | .018 |
|  | Univariate OR (99 CI) | 1.0 | 1.13 (0.69, 1.84) | 1.40 (0.91, 2.14) | 1.27 (0.71, 2.27) | 1.79 (0.98, 3.26) | 1.45 (0.81, 2.59) | .006 |

Supplementary Table 7. Dose Dependent Associations between Electronic Device use and Catch Up Sleep including raw frequencies, univariate and multivariate associations.

|  | Quintiles N (%) | Device Absent, Never Use | Device Present, Never Use | Rarely | A Few nights/month | A Few nights/ week | Every or almost every night | P for Trend |
| --- | --- | --- | --- | --- | --- | --- | --- | --- |
| Computer | Normal 3 Quintiles 701 (59.2) | 161 (59.0) | 57 (57.6) | 129 (61.7) | 85 (57.8) | 135 (61.6) | 134 (56.5) |  |
|  | Abnormal 2 Quintiles 483 (40.8) | 112 (41.0) | 42 (42.4) | 80 (38.3) | 62 (42.2) | 84 (38.4) | 103 (43.5) |  |
|  | Multivariate OR (99 CI) | 1.0 | 1.00 (0.54, 1.87) | 0.85 (0.52, 1.39) | 0.99 (0.58, 1.72) | 0.81 (0.49, 1.32) | 1.00 (0.62, 1.62) | 0.73 |
|  | Univariate OR (99 CI) | 1.0 | 1.06 (0.57, 1.95) | 0.89 (0.55, 1.45) | 1.05 (0.61, 1.79) | 0.89 (0.56, 1.44) | 1.11 (0.70, 1.76) | 0.81 |
| Mobile | Normal 3 Quintiles 701 (59.2) | 111 (66.9) | 75 (56.0) | 129 (62.0) | 68 (56.2) | 126 (58.3) | 192 (56.6) |  |
|  | Abnormal 2 Quintiles 483 (40.8) | 55 (33.1) | 59 (44.0) | 79 (38.0) | 53 (43.8) | 90 (41.7) | 147 (43.4) |  |
|  | Multivariate OR (99 CI) | 1.0 | 1.55 (0.83, 2.90) | 1.12 (0.63, 1.99) | 1.44 (0.75, 2.76) | 1.29 (0.73, 2.27) | 1.31 (0.77, 2.25) | 0.41 |
|  | Univariate OR (99 CI) | 1.0 | 1.59 (0.86, 2.94) | 1.24 (0.71, 2.17) | 1.57 (0.83, 2.97) | 1.44 (0.83, 2.51) | 1.55 (0.93, 2.57) | 0.07 |
| TV | Normal 3 Quintiles 701 (59.2) | 309 (58.9) | 30 (63.8) | 130 (56.5) | 65 (60.7) | 96 (60.0) | 71 (61.7) |  |
|  | Abnormal 2 Quintiles 483 (40.8) | 216 (41.1) | 17 (36.2) | 100 (43.5) | 42 (39.3) | 64 (40.0) | 44 (38.3) |  |
|  | Multivariate OR (99 CI) | 1.0 | 0.87 (0.38, 2.00) | 1.11 (0.73, 1.69) | 0.88 (0.50, 1.56) | 0.97 (0.60, 1.57) | 0.89 (0.51, 1.55) | 0.64 |
|  | Univariate OR (99 CI) | 1.0 | 0.81 (0.36, 1.83) | 1.10 (0.73, 1.66) | 0.92 (0.53, 1.62) | 0.95 (0.59, 1.53) | 0.89 (0.51, 1.53) | 0.64 |
| Radio | Normal 3 Quintiles 701 (59.2) | 321 (63.2) | 89 (54.6) | 126 (56.0) | 54 (53.5) | 43 (48.3) | 68 (69.4) |  |
|  | Abnormal 2 Quintiles 483 (40.8) | 187 (36.8) | 74 (45.4) | 99 (44.0) | 47 (46.5) | 46 (51.7) | 30 (30.6) |  |
|  | Multivariate OR (99 CI) | 1.0 | 1.41 (0.87, 2.26) | 1.32 (0.86, 2.02) | 1.39 (0.78, 2.45) | 1.85 (1.01, 3.38) | 0.71 (0.38, 1.32) | 0.48 |
|  | Univariate OR (99 CI) | 1.0 | 1.43 (0.89, 2.28) | 1.35 (0.89, 2.05) | 1.49 (0.85, 2.63) | 1.84 (1.01, 3.33) | .076 (0.41, 1.40) | 0.31 |

Supplementary Table 8. Dose Dependent Associations between Electronic Device use and Wake Lag including raw frequencies, univariate and multivariate associations.

|  | Quintiles N (%) | Device Absent, Never Use | Device Present, Never Use | Rarely | A Few nights/month | A Few nights/ week | Every or almost every night | P for Trend |
| --- | --- | --- | --- | --- | --- | --- | --- | --- |
| Computer | Normal 3 Quintiles 754 (63.7) | 75 (75.8) | 189 (69.2) | 130 (62.2) | 97 (66.0) | 131 (59.8) | 132 (55.7) |  |
|  | Abnormal 2 Quintiles 430 (36.3) | 24 (24.2) | 84 (30.8) | 79 (37.8) | 50 (34.0) | 88 (40.2) | 105 (44.3) |  |
|  | Multivariate OR (99 CI) | 1.0 | 0.65 (0.32, 1.33) | 1.30 (0.78, 2.16) | 1.05 (0.59, 1.87) | 1.33 (0.80, 2.21) | 1.56 (0.95, 2.55) | .005 |
|  | Univariate OR (99 CI) | 1.0 | 0.72 (0.36, 1.44) | 1.37 (0.83, 2.25) | 1.16 (0.66, 2.03) | 1.51 (0.93, 2.47) | 1.79 (1.11, 2.88) | <.001 |
| Mobile | Normal 3 Quintiles 754 (63.7) | 96 (71.6) | 125 (75.3) | 148 (71.2) | 77 (63.6) | 129 (59.7) | 179 (52.8) |  |
|  | Abnormal 2 Quintiles 430 (36.3) | 38 (28.4) | 41 (24.7) | 60 (28.8) | 44 (36.4) | 87 (40.3) | 160 (47.2) |  |
|  | Multivariate OR (99 CI) | 1.0 | 1.13 (0.57, 2.26) | 1.12 (0.60, 2.08) | 1.53 (0.77, 3.04) | 1.82 (1.00, 3.32) | 2.23 (1.26, 3.94) | <.001 |
|  | Univariate OR (99 CI) | 1.0 | 1.21 (0.61, 2.38) | 1.24 (0.67, 2.27) | 1.74 (0.89, 3.41) | 2.06 (1.15, 3.69) | 2.73 (1.59, 4.68) | <.001 |
| TV | Normal 3 Quintiles 754 (63.7) | 28 (59.6) | 363 (69.1) | 143 (62.2) | 58 (54.2) | 97 (60.6) | 65 (56.5) |  |
|  | Abnormal 2 Quintiles 430 (36.3) | 19 (40.4) | 162 (30.9) | 87 (37.8) | 49 (45.8) | 63 (39.4) | 50 (43.5) |  |
|  | Multivariate OR (99 CI) | 1.0 | 1.52 (0.67, 3.45) | 1.31 (0.85, 2.02) | 1.70 (0.96, 2.99) | 1.39 (0.85, 2.27) | 1.62 (0.93, 2.82) | .005 |
|  | Univariate OR (99 CI) | 1.0 | 1.52 (0.68, 3.40) | 1.36 (0.89, 2.09) | 1.89 (1.09, 3.30) | 1.46 (0.90, 2.36) | 1.72 (1.00, 2.96) | .001 |
| Radio | Normal 3 Quintiles 754 (63.7) | 105 (64.4) | 347 (68.3) | 131 (58.2) | 60 (59.4) | 48 (53.9) | 63 (64.3) |  |
|  | Abnormal 2 Quintiles 430 (36.3) | 58 (35.6) | 161 (31.7) | 94 (41.8) | 41 (40.6) | 41 (46.1) | 35 (35.7) |  |
|  | Multivariate OR (99 CI) | 1.0 | 1.13 (0.69, 1.85) | 1.50 (0.97, 2.31) | 1.35 (0.75, 2.42) | 1.86 (1.01, 3.42) | 1.07 (0.58, 1.97) | .043 |
|  | Univariate OR (99 CI) | 1.0 | 1.19 (0.73, 1.94) | 1.55 (1.01, 2.37) | 1.47 (0.83, 2.62) | 1.84 (1.01, 3.36) | 1.20 (0.66, 2.17) | .013 |
